# Ultrasonic defect characterization using parametric-manifold mapping

**DOI:** 10.1098/rspa.2017.0056

**Published:** 2017-06-07

**Authors:** A. Velichko, L. Bai, B. W. Drinkwater

**Affiliations:** Department of Mechanical Engineering, University of Bristol, Queens Building, University Walk, Bristol BS8 1TR, UK

**Keywords:** ultrasonic non-destructive testing, defect characterization, parametric manifold

## Abstract

The aim of ultrasonic non-destructive evaluation includes the detection and characterization of defects, and an understanding of the nature of defects is essential for the assessment of structural integrity in safety critical systems. In general, the defect characterization challenge involves an estimation of defect parameters from measured data. In this paper, we explore the extent to which defects can be characterized by their ultrasonic scattering behaviour. Given a number of ultrasonic measurements, we show that characterization information can be extracted by projecting the measurement onto a parametric manifold in principal component space. We show that this manifold represents the entirety of the characterization information available from far-field harmonic ultrasound. We seek to understand the nature of this information and hence provide definitive statements on the defect characterization performance that is, in principle, extractable from typical measurement scenarios. In experiments, the characterization problem of surface-breaking cracks and the more general problem of elliptical voids are studied, and a good agreement is achieved between the actual parameter values and the characterization results. The nature of the parametric manifold enables us to explain and quantify why some defects are relatively easy to characterize, whereas others are inherently challenging.

## 1. Introduction

The aim of ultrasonic non-destructive evaluation and structural health monitoring includes the detection and characterization of defects [1–5], and an understanding of the nature of defects is essential for the assessment of structural integrity in safety critical systems [6]. There are several types of defects that can potentially occur in a structure, whose effects need to be treated differently. Cracks are commonly regarded as ‘the defects of most concern’ [7] because they can lead to rapid growth and hence failure of a structure [8]. For this reason, characterization of cracks has been studied extensively in the literature, including measurements based on single element transducers [9,10] and transducer arrays [11,12]. The proposed approaches include measuring the scattered amplitude [10], the time-of-flight diffraction technique [13], image-based characterization [11,14] and characterization using the scattering matrix [11,12], which gives a far-field scattering amplitude for every combination of incident and scattered directions.

Once a defect has been non-destructively detected, the next requirement is to characterize it and hence to discover its physical nature. In the absence of any characterization information, the worst case scenario is assumed, which is often to classify the detected defect as an unfavourably (w.r.t. the loading) oriented surface-breaking crack. Here, the worst case defect is one that will most rapidly result in a failure of the structure. Improved defect characterization information allows the worst case scenario to be replaced by a more accurate representation of reality. Of course, in some cases the characterization may confirm the worst case scenario, but in others the detected defects can be relatively benign, for example rounded pores or inclusions introduced in the manufacturing process. Whatever the characterization result, the outcome of more accurate characterization is a better prediction of the remaining life of a structure.

There are two main approaches to the defect characterization problem seen in the literature. The first attempts to reconstruct the defect geometry without any preliminary assumptions about the defect. For example, an iterative method can be applied, in which a defect geometry is iteratively updated and a forward scattering model is used to calculate a scattering matrix until a match with the measured data is reached [15]. In some cases a model-based inversion, which makes use of an approximate analytical expression for the forward scattering problem, can be applied. This approach is widely used in guided waves tomography for the reconstruction of the thickness map of corrosion damage [16]. Alternatively, semi-analytical inversion schemes have been developed [17]. However, a regularization procedure is required in general inversion approaches [18] in order to deal with the ill-posedness of the studied problem, and issues including numerical stability and convergence need to be addressed [19]. This has led researchers to consider more targeted approaches, and the second approach makes use of the fact that there is only a limited number of possible defect types that can occur in practice. Each defect type can then be efficiently described by a limited set of parameters. In this case, the characterization problem becomes one of estimating this smaller set of defect parameters from the measured scattering data. Typically, the method of solution adopted is to form a large database from many forward simulations and then compare experimental data with that in the database, i.e. the database is searched and the closest match is used to characterize the defect. In these database search methods, use can be made of the vast body of the literature on classification algorithms and their applications [20–24]. Neural networks and the support vector machine are examples of the widely used classification approaches. Their use can be found in a range of applications, including radar target recognition [25], underwater target classification [26], classification of electroencephalogram signals [27] and in bioinformatics (e.g. gene selection for cancer classification [28]). Using this classification algorithm approach to the characterization problem, good results have been achieved on simulated data and in idealized experiments containing machined notches [12] and volumetric elliptical voids [29]. In addition, the effect of coherent grain noise on these classification schemes was explored [30].

However, there is a philosophical problem with the use of the above classification approach—the classification algorithms are ‘black-boxes’ completely defined by the training data. In addition, the classification approach relies on subjective choices such as the defect class definition and the number of defect classes, which limits the generalizability of the approach, especially when three or more parameters need to be determined from the inversion process. In this paper, a new defect characterization method is proposed to address the above issues. The crucial observation is that the defect database can be represented by a parametric manifold in the measurement domain, and can be approximated to any resolution with a finite number of training samples using interpolation schemes. As such, any characterization procedure is fully determined by the shape of the parametric manifold, which also determines the achievable characterization accuracy.

The proposed approach has some important practical benefits. Firstly, the characterization is performed in the principal component domain. In this case, only a few largest principal components can be taken, which resolves the issues related to high dimensional characterization spaces. Secondly, the manifold representation of the parametric defect space provides a much more intuitive/insightful geometric understanding and helps to ‘visualize’ the defect characterization problem. The proposed method naturally handles any measurement scheme (e.g. arrays, multiple probes and scanning) and allows us to consider different defect types. Thirdly, in practice, the available measurement information is always limited, and, in this case, an estimation of the characterization uncertainty is critical. The proposed approach naturally introduces the method for the characterization uncertainty estimation, so the final characterization result is represented by the probability density distribution in the defect parameter space.

Here, we focus our efforts on the use of an ultrasonic array to characterize two different defect types: surface-breaking cracks and elliptical voids. All defects are assumed to be two dimensional and a one-dimensional linear ultrasonic array is used to perform relevant measurements. However, it should be stressed that the characterization procedure proposed in the paper is independent of the dimensionality of defects and potentially can be directly applied to characterize three-dimensional defects. As stated above, cracks are detrimental to structural integrity, and the detection and characterization of surface-breaking cracks is of particular industrial interest. Volumetric voids (pores) are also important because they can be potential initiation sites of cracks [31], and ellipses provide a reasonable simplification of a wide range of volumetric voids. Surface-breaking cracks can be described by two parameters: size and orientation angle. For elliptical voids, besides size and orientation angle, a third parameter—aspect ratio—is needed to define the shape. Hence, the characterization of elliptical voids naturally extracts the aspect ratio, which is a measure of sharpness and could be useful in fatigue life predictions.

## 2. Defect characterization problem

**Figure 1.**
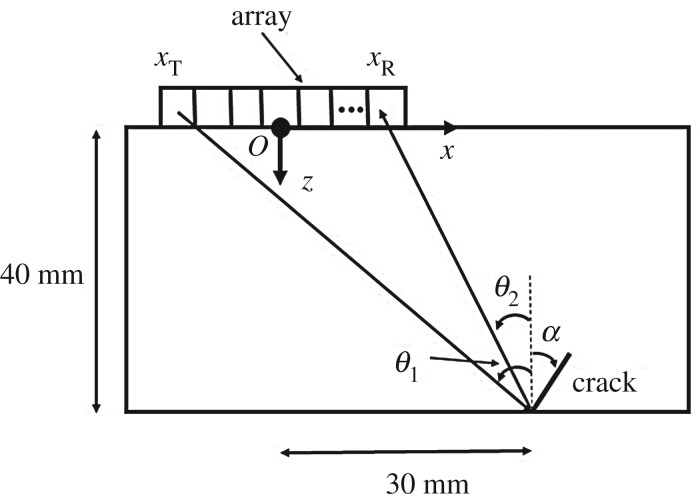
Array measurement configuration in simulation and experiments (for surface-breaking cracks).

Figure 1 shows a typical ultrasonic array inspection configuration for surface-breaking cracks located on the back face of a flat plate. The incident and scattering angles and the orientation angle of surface-breaking cracks are all defined with respect to the back surface normal, and are positive if measured clockwise. In the example shown here, the material is aluminium (Young’s modulus, 69 GPa; Poisson’s ratio, 0.334; density, 2700 kg m^−3^), and the thickness of the test specimen is 40 mm. A 2.5 MHz, 64-element array is used as an example throughout this paper, and the element pitch of the array is 0.50 mm (i.e. 0.2λ at the centre frequency). In figure 1, the array is moved away from the crack by 30 mm, because otherwise the crack would be obscured by the high-intensity reflection from the back wall in the image.

Firstly, defects with idealized geometries are considered, so the cracks are assumed to be smooth. In this case, each surface-breaking crack is fully characterized by two parameters—length and orientation angle. In reality, all cracks have some level of roughness, which is usually described by RMS height, *σ*_rough_, and correlation length, λ_rough_ [32]. However, if the roughness level is not too high, so that the RMS height is much smaller than the ultrasonic wavelength, then the geometry of the crack can still be approximately described by its length and orientation angle. The defect characterization problem can then be formulated as estimating the crack parameters from the ultrasonic array measurements.

From the defect characterization point of view, the information that can be extracted from the ultrasonic array data is the scattering behaviour of the defect. The scattering information can be represented in the form of a scattering matrix [11]. More specifically, for a unit amplitude plane incident wave propagating in the direction *θ*_in_, the scattered wave in the far field of the defect at the direction *θ*_sc_ is given by

2.1 $$u_{\mathrm{sc}}=\sqrt{\frac{\lambda }{r}}S(\theta_{\mathrm{in}},\theta_{\mathrm{sc}},\omega )\;e^{ikr},$$

where λ is the ultrasonic wavelength, *k*=2*π*/λ is the wavenumber, *ω* is the angular frequency, *r* is the distance from the nominal defect centre and *S* is the scattering matrix. Note that the proposed characterization method does not depend on the particular testing procedure used to perform measurements. To illustrate the performance of the characterization method simple direct contact measurements were used. In this case, the scattering information extracted from the ultrasonic array data corresponds to the longitudinal waves, and shear waves and mode conversion effects are not considered. However, the proposed approach is generally applicable to any experimental configuration and wave mode type.

**Figure 2.**
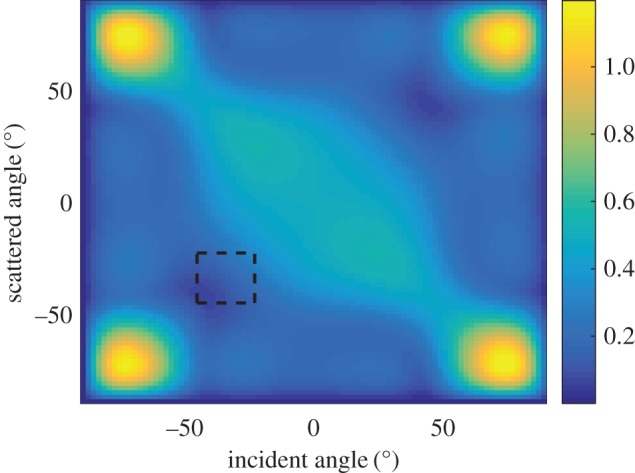
The scattering matrix of a 1λ, 0° surface-breaking crack. The dashed box represents the angular range extractable from the measurement configuration shown in figure 1. (Online version in colour.)

The scattering matrices of surface-breaking cracks were simulated using an efficient finite-element model [33], and figure 2 shows the scattering matrix of a 1λ, 0° surface-breaking smooth crack. Although the scattering matrix is shown for the range of [−90°,90°], the maxima around the incident and scattered angles of ±90° are not extractable for the example shown in figure 1 because of the finite size of the array aperture. The dashed box in figure 2 represents the angular range extractable from the measurement configuration shown in figure 1. In general, the scattering matrix is a complex-valued function; however, its phase depends on the position of the nominal centre of the defect and, as this is not known *a priori*, it is difficult to extract the phase from the measurements. Therefore, in this paper only the amplitude of the scattering matrix is considered. However, it is noted that further information is contained in the phase part of the scattering matrix and future work could look at how to make use of this reliably.

## 3. Defect manifold

### (a) Principal component analysis

The defect characterization problem consists of estimating the defect parameters from the extracted components of the scattering matrix. However, before the characterization procedure is developed, we explore the general structure of the set of the scattering matrices for a certain defect type.

Let us consider the set of all possible scattering matrices for a particular measurement configuration

3.1 $$S=\{S(\theta_{\mathrm{in}},\theta_{\mathrm{sc}},\omega ),\;\theta_{\mathrm{in},1}\leq \theta_{\mathrm{in}}\leq \theta_{\mathrm{in},2},\;\theta_{\mathrm{sc},1}\leq \theta_{\mathrm{sc}}\leq \theta_{\mathrm{sc},2},\;\omega_{1}\leq \omega \leq \omega_{2}\},$$

which will be referred to as the *s*-space.

For any defect characterization procedure, some metric is required to quantify the distance between a measurement and the entire *s*-space. The distance (or similarity) between two scattering matrices can be measured in many different ways. For example, the *L*_2_ (or Euclidean) norm can be used as

3.2 $$\begin{array}{rl} ∥S_{1}-S_{2}∥ & =\sqrt{{\left(S_{1}-S_{2},S_{1}-S_{2}\right)}_{L_{2}}},\\ {\left(S_{1},S_{2}\right)}_{L_{2}} & =\int S_{1}S_{2}\;d\theta_{\mathrm{in}}\;d\theta_{\mathrm{sc}}\;d\omega . \end{array}\}$$

In practice, the scattering matrix can be measured at a finite number of incident and scattered angles and frequencies only. For simplicity, it is assumed that the measurement angles, *θ*_in,*n*_,*θ*_sc,*m*_, and frequencies, *ω*_*k*_, are equally spaced and there are *N*_in_ incident angles, *N*_sc_ scattered angles and *N*_f_ frequencies. In this case, each scattering matrix *S* can be considered as a vector **s**={*s*_1_,…,*s*_*N*_*s*__}^T^, *s*_*l*_=*S*(*θ*_in,*n*_,*θ*_sc,*m*_,*ω*_*k*_) in an *N*_*s*_=*N*_in_×*N*_sc_×*N*_f_-dimensional space.

For the typical ultrasonic array inspection *N*_in_,*N*_sc_,*N*_f_∼10, therefore, the dimension of the *s*-space is *N*_*s*_∼10^3^. The analysis of data in such high dimensional space is difficult. However, in many cases each scattering matrix can be efficiently described by much fewer components. One possibility to reveal this reduction in dimensionality is to apply principal component analysis (PCA) [34,35] to the scattering matrices. PCA takes into account the variability of data in different directions, and chooses a new coordinate system, so each point in the manifold is represented with fewer coordinates, which are referred to as the principal components hereafter.

The class of defects that can be parametrically described by *N*_*p*_ parameters **p**={*p*_1_,…,*p*_*Np*_}, *p*_1*n*_≤*p*_*n*_≤*p*_2*n*_, *n*=1,…,*N*_*p*_, is now considered. The corresponding set of scattering matrices is defined as *S*_*p*_=*S*(*θ*_in_,*θ*_sc_,*ω*;**p**). The set of scattering matrices in the defect class can be represented by the *N*_*s*_×*M* matrix ${\text{S}}_{p}=\{{\text{s}}_{p}({\text{p}}_{1})-{\bar{\text{s}}}_{p},\ldots ,{\text{s}}_{p}({\text{p}}_{M})-{\bar{\text{s}}}_{p}\}\in R^{N_{s}\times M}$, where *M* is the total number of scattering matrices in the defect class, vector **s**_*p*_ corresponds to the defect scattering matrix *S*_*p*_ and ${\bar{\text{s}}}_{p}$ is the vector corresponding to the mean scattering matrix of the defect class. The new coordinate system is obtained by singular value decomposition of the covariance matrix of **S**_*p*_

3.3 $$\text{R}=\text{V}\text{D}{\text{V}}^{T},$$

where **R** is the *N*_*s*_×*N*_*s*_ covariance matrix of **S**_*p*_ and can be obtained as

3.4 $$\text{R}=\frac{1}{M-1}{\text{S}}_{p}{\text{S}}_{p}^{T}.$$

The diagonal matrix **D** contains the eigenvalues of **R**. The coordinate axes of the new coordinate system are now given by the column vectors of **V**, so any scattering matrix, **s**, in the new coordinate system is represented by the vector **s**^(pc)^ as

3.5 $${\text{s}}^{\left(\mathrm{pc}\right)}={\text{V}}^{T}(\text{s}-{\bar{\text{s}}}_{p}).$$

After the application of PCA, the defect class is effectively embedded in a lower dimensional space (which we call principal component space, or pc-space), since the first *N*_pc_ coordinates account for most of the variation of the set *S*_*p*_. Normally, we have *N*_pc_≪*N*_*s*_, and the value of *N*_pc_ can be determined, for example, by setting some threshold as

3.6 $$\frac{d_{n}}{\max d_{n}}\geq d_{0},\;n=1,\ldots ,N_{\mathrm{pc}},$$

where *d*_*n*_ are the eigenvalues of covariance matrix **R** and *d*_0_ is the threshold. Therefore, only principal components with eigenvalues greater than the threshold are retained.

### (b) Structure of defect manifold

The important observation is that the set of defect class scattering matrices represents an *N*_p_-dimensional manifold in the pc-space. This manifold will be referred to as the defect manifold or *d*-manifold. The structure of the *d*-manifold provides a fundamental insight into the defect characterization problem and determines the achievable characterization accuracy.

**Figure 3.**
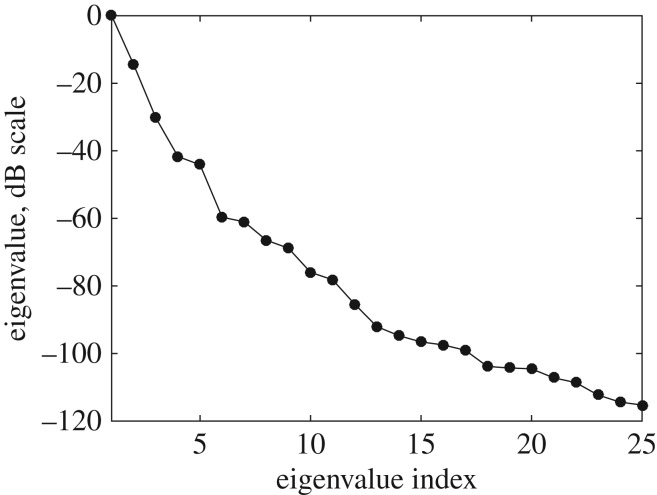
Eigenvalues of the covariance matrix (25 largest eigenvalues) for surface-breaking cracks.

**Figure 4.**
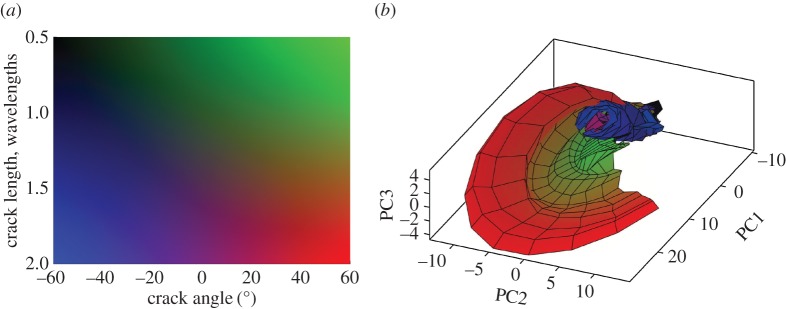
Surface-breaking cracks: (*a*) parameter space; (*b*) *d*-manifold in three-dimensional pc-space.

For the example configuration considered in §2 (figure 1), the defect parameter space is defined as **p**={*θ*_crack_,*a*_crack_}, −60°≤*θ*_crack_≤60°, 0.5λ≤*a*_crack_≤2λ, where *θ*_crack_ is the crack orientation and *a*_crack_ is the crack length. The scattering matrices were calculated on a uniform grid in the parameter space with the parameter steps Δ*θ*_crack_=2°, Δ*a*_crack_=0.1λ. Figure 3 shows the first 25 largest eigenvalues of the covariance matrix **R**. The threshold of *d*_0_=0.005 was taken, and in this case *N*_pc_=5. Figure 4*a*,*b* shows the parameter *p*-space and the shape of the *d*-manifold in three-dimensional pc-space, respectively. Each point on the defect manifold corresponds to some particular point in the defect parameter space. To visualize this mapping, a colour map in the defect parameter space was used, and the same colour map was then used to plot the *d*-manifold. In other words, the same colours in the defect parameter space and the defect manifold correspond to one particular set of defect parameters. It can be seen (by observing the red region in figure 4) that, for crack parameters λ≤*a*_crack_≤2λ, 40°≤*θ*_crack_≤60°, this surface is well resolved, suggesting that in this region there is enough information for unique defect characterization. Note that, at these crack orientation angles, the specular reflection from the crack can be detected by the array, and, therefore, the measured part of the scattering matrix contains its maximum peak. For other parameters of the crack the shape of the *d*-manifold is more complex, which can be explained by the following factors. Firstly, the corresponding regions of the *d*-manifold are defined by more than three principal components and cannot be visualized in the three-dimensional pc-space. Secondly, in this case, the specular reflection from the crack is not detected by the array, and the measured part of scattering matrix is less sensitive to the crack parameters. Therefore, the points **s**^(pc)^, which represent the scattering matrices in pc-space, are located close to each other and hence defect characterization is more challenging.

We can now explore the global structure of the *d*-manifold. The distance between two scattering matrices *S*_*p*1_=*S*_*p*_(**p**_1_), *S*_*p*2_=*S*_*p*_(**p**_2_) can be measured in pc-space using the *L*_2_ norm defined by (3.2) as

3.7 $$∥S_{p1}-S_{p2}{∥}_{\mathrm{pc}}=\sqrt{\left({\text{s}}_{p1}^{\left(\mathrm{pc}\right)}-{\text{s}}_{p2}^{\left(\mathrm{pc}\right)},{\text{s}}_{p1}^{\left(\mathrm{pc}\right)}-{\text{s}}_{p2}^{\left(\mathrm{pc}\right)}\right)}.$$

Alternatively, the distance metric in pc-space leads to a corresponding distance metric on the *d*-manifold. Then, an alternative distance measure between two scattering matrices *S*_*p*1_, *S*_*p*2_ can be taken as the length of the geodesic line between ${\text{s}}_{p1}^{\left(\mathrm{pc}\right)}$ and **s**^(pc)^_*p*2_ on the *d*-manifold, or the minimum length of all possible paths between **s**^(pc)^_*p*1_ and ${\text{s}}_{p2}^{\left(\mathrm{pc}\right)}$ on the *d*-manifold,

3.8 $$∥S_{p1}-S_{p2}{∥}_{d}=\min\int_{{\text{s}}_{p1}^{\left(\mathrm{pc}\right)}}^{{\text{s}}_{p2}^{(\mathrm{pc})}}|d{\text{s}}_{p}^{\left(\mathrm{pc}\right)}|.$$

**Figure 5.**
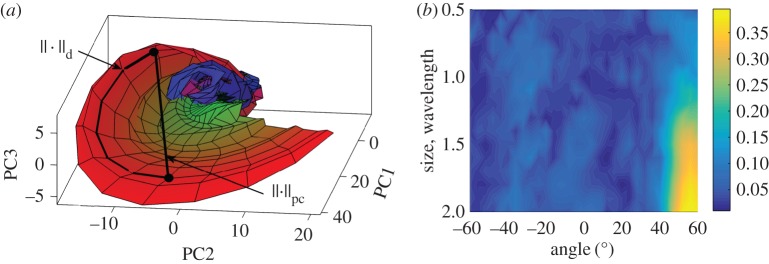
(*a*) Schematic of distance metric in the pc-space, ∥⋅∥_pc_, and on the *d*-manifold, ∥⋅∥_*d*_; (*b*) map of the *d*-index for the surface-breaking cracks. (Online version in colour.)

The physical meaning of the metric (3.8) is illustrated in figure 5*a*. The *d*-manifold distance metric is given by the length of the geodesic line (the shortest distance) connecting two points on the *d*-manifold. It can be seen that the manifold distance (3.8) between any two scattering matrices is always greater than the Euclidean distance (3.7) between the same scattering matrices in pc-space, ∥⋅∥_*d*_≥∥⋅∥_pc_. Then, the geometry of the *d*-manifold can be characterized by the following dimensionless parameter *I*_*d*_, which is referred to as the *d*-index:

3.9 $$I_{d}(\text{p})=\min_{\text{q}}\frac{∥S_{p}(\text{p})-S_{p}(\text{q}){∥}_{\mathrm{pc}}}{∥S_{p}(\text{p})-S_{p}(\text{q}){∥}_{d}},$$

where **p**,**q** are parameter vectors from the defect parameter space. The *d*-index is always less than 1, 0≤*I*_*d*_≤1, and characterizes the stability of the characterization result. Qualitatively, a small value of the *d*-index at the point **p** means that there exists another parameter vector **q** which is not located in the vicinity of **p**, but the scattering matrices *S*_*p*_(**p**) and *S*_*p*_(**q**) are very similar to each other. Therefore, at such parameter points the defect characterization will be sensitive to noise and unique defect characterization could be difficult.

Figure 5*b* shows the *d*-index of the defect manifold as a function of surface-breaking crack parameters (orientation angle and crack length). The calculations of (3.8) were performed using Dijkstra’s algorithm [36]. It can be seen that in the region λ≤*a*_crack_≤2λ, 40°≤*θ*_crack_≤60° of the parameter space the *d*-index is high, suggesting that the characterization result will be stable with respect to noise. However, characterization in all other points will be more sensitive to noise and, therefore, more uncertain. Note that this result agrees with the three-dimensional shape of the *d*-manifold shown in figure 4*b*, but also allows us to quantitatively characterize the structure of the *d*-manifold in higher dimensional spaces.

## 4. Defect characterization method

Note that each defect class consists of a database of defects with idealized geometries, for example straight cracks with different lengths and orientations. An experimentally measured scattering matrix always contains some noise compared with the scattering matrices in the defect database. This noise can be a random experimental noise, but also coherent noise which is caused by the differences between real defect geometries and those of the defect in the database, for example surface roughness of the crack. There are also a number of other sources of coherent noise apart from the defect roughness. For example, noise can arise from interference from other defects and structural features located near the defect of interest. This noise is the result of imperfect focusing due to the finite array aperture and the diffraction limit. Another possible reason for coherent noise is the limitations of the two-dimensional model of the ultrasonic array and defect scattering. In any case, this means that, in practice, it will be impossible to achieve an exact match between an experimentally extracted scattering matrix and the scattering matrices from some idealized defect class.

Based on the analysis performed above, it can be seen that any experimental scattering matrix can be represented by a vector **s**^(pc)^_exp_ in pc-space. However, in general, this vector is not necessarily located on the *d*-manifold of the defect class because of the combined effects of random and coherent experimental noise. This can be expressed as

4.1 $${\text{s}}_{\exp}^{(\mathrm{pc})}={\text{s}}_{p}^{\left(\mathrm{pc}\right)}+{\text{n}}_{p}^{\left(\mathrm{pc}\right)},$$

where the vector ${\text{s}}_{p}^{\left(\mathrm{pc}\right)}$ corresponds to the scattering matrix of the defect with the parameter **p** and **n**^(pc)^_*p*_ represents a noise perturbation of this scattering matrix in pc-space.

It is assumed that the noise is described by some statistical model, so the noise vector ${\text{n}}_{p}^{\left(\mathrm{pc}\right)}$ in expression (4.1) represents one particular realization of the noise. Then, the defect characterization problem can be formulated in the following way: given the occurrence of the measured scattering matrix **s**^(pc)^_exp_, what is the probability that this scattering matrix can be represented by the defect parameter **p**? Note that the defect parameter **p** represents a continuous variable. Therefore, the probability is given by *ρ*_*p*_(**p** | **s**^(pc)^_exp_)*dp*, where *ρ*_*p*_(**p** | **s**^(pc)^_exp_) is the conditional probability density function of the defect parameter **p** and *dp*=*Δp*_1_×⋯×*Δp*_*N*_*p*__ is a volume element in parameter space.

Alternatively, this question can be formulated in a different form: given the defect parameter **p**, what is the probability that, for some particular noise realization, the perturbed scattering matrix ${\text{s}}_{p}^{(\mathrm{pc})}+{\text{n}}_{p}^{\left(\mathrm{pc}\right)}$ will match the experimental measurements ${\text{s}}_{\exp}^{\left(\mathrm{pc}\right)}$? Similar to the previous case, this probability is described by $\rho_{S}({\text{s}}_{\exp}^{\left(\mathrm{pc}\right)}\;|\;\text{p})ds^{\left(\mathrm{pc}\right)}$, where *ρ*_*S*_(**s**^(pc)^_exp_ | **p**) is the conditional probability density function of the vector **s**^(pc)^_exp_ in pc-space and *ds*^(pc)^ is a volume element in pc-space.

It is easy to show that the probability density functions *ρ*_*p*_(**p** | **s**^(pc)^_exp_) and *ρ*_*S*_(**s**^(pc)^_exp_ | **p**) are directly related to each other. According to Bayes’ theorem, the posterior probability $\rho_{p}(\text{p}\;|\;{\text{s}}_{\exp}^{\left(\mathrm{pc}\right)})$ is given by

4.2 $$\rho_{p}(\text{p}\;|\;{\text{s}}_{\exp}^{\left(\mathrm{pc}\right)})=\rho_{S}({\text{s}}_{\exp}^{\left(\mathrm{pc}\right)}\;|\;\text{p})\frac{\rho_{p}(\text{p})}{\rho_{S}({\text{s}}_{\exp}^{\left(\mathrm{pc}\right)})},$$

where *ρ*_*p*_(**p**) is the marginal probability density distribution of the defect parameters **p** and *ρ*_*S*_(**s**^(pc)^_exp_) is the marginal probability distribution of the measured scattering matrix. In the rest of this paper, it is assumed for simplicity that all defect parameters from the defect class and all possible measured scattering matrices are equally probable. In other words, probability distributions *ρ*_*p*_(**p**) and *ρ*_*S*_(**s**^(pc)^_exp_) are uniform. In this case

4.3 $$\rho_{p}(\text{p}\;|\;{\text{s}}_{\exp}^{\left(\mathrm{pc}\right)})=C\rho_{S}({\text{s}}_{\exp}^{\left(\mathrm{pc}\right)}\;|\;\text{p}),$$

where the normalization constant *C* is given by integration of the conditional probability over the parameter space

4.4 $$C={\left(\int \rho_{S}({\text{s}}_{\exp}^{(\mathrm{pc})}\;|\;\text{p})\;dp\right)}^{-1}.$$

Note that in a given industrial inspection some additional information might be available about possible defect types and defect parameters. This information can be naturally incorporated into the characterization procedure by using non-uniform marginal probability density distributions of the defect parameters. Once the probability density function is estimated, the characterization result is given by the defect parameters **p**_*c*_ with the highest probability $\rho_{p}(\text{p}\;|\;{\text{s}}_{\exp}^{\left(\mathrm{pc}\right)})$.

However, practically it is more convenient to calculate the probability density function $\rho_{S}({\text{s}}_{\exp}^{\left(\mathrm{pc}\right)}\;|\;\text{p})$ at each defect parameter point **p** first, and then calculate the function *ρ*_*p*_(**p** | **s**^(pc)^_exp_) using expressions (4.3) and (4.4). The conditional probability density function *ρ*_*S*_(**s**^(pc)^ | **p**) as a function of the vector **s**^(pc)^ describes the distribution of noise in pc-space. This function can be estimated from an experiment, or calculated using some assumed noise model. In any case, the defect characterization result and the corresponding confidence level depend on the choice of the noise model. Therefore, the knowledge of *ρ*_*S*_(**s**^(pc)^ | **p**) is critical for defect characterization.

Generally, the maximum probability point **p**_*c*_ has to be estimated numerically. However, one important case when this point can be easily found is for isotropic noise distribution in pc-space. This means that the probability density function *ρ*_*S*_(**s**^(pc)^ | **p**) depends on the distance between the experimental (noisy) scattering matrix **s**^(pc)^ and the scattering matrix **s**^(pc)^_*p*_ of the defect with parameter **p** only. If in addition the probability density function *ρ*_*S*_(**s**^(pc)^ | **p**) is independent of the parameter point **p**, then

4.5 $$\rho_{S}({\text{s}}^{(\mathrm{pc})}\;|\;\text{p})=h(|{\text{s}}^{\left(\mathrm{pc}\right)}-{\text{s}}_{p}^{\left(\mathrm{pc}\right)}|).$$

The most probable defect parameter **p**_*c*_ corresponds to the point **s**^(pc)^_*p*_*c*__ on the *d*-manifold. If the function *h*(*r*) monotonically decays as r→∞, then the point ${\text{s}}_{p_{c}}^{\left(\mathrm{pc}\right)}$ corresponds to the nearest point or simply the projection of the testing point **s**^(pc)^_exp_ onto the *d*-manifold. It is possible to derive a semi-analytic expression for the projection point on the *d*-manifold and the correspond result is given in appendix Aa. Finally, the characterization parameter vector **p**_*c*_ can be found by mapping the point **s**^(pc)^_*p*_*c*__ back into defect parameter space.

## 5. General model of coherent noise

It has been noted earlier that the conditional probability density function *ρ*_*S*_(**s**^(pc)^ | **p**) describes the distribution of noise in pc-space. This function defines the defect characterization result and, therefore, plays a central part in the defect characterization procedure. Practically, the noise distribution can be estimated from experimental testing under realistic measurement conditions. However, this approach requires many samples with real defects in order to measure the statistics of the noise. Alternatively, the function *ρ*_*S*_(**s**^(pc)^ | **p**) can be simulated using some assumed noise model. The difficulty is that, in general, there are many different factors which contribute to the noise. Detailed analysis of the different noise models is beyond the scope of this paper and will be performed in a separate publication. Here, we just note that usually random noise can be suppressed by averaging and also the PCA acts as an additional filter [12]. Therefore, in practice, the coherent noise makes the main contribution to the total noise level. In this section, a general coherent noise model is proposed.

**Figure 6.**
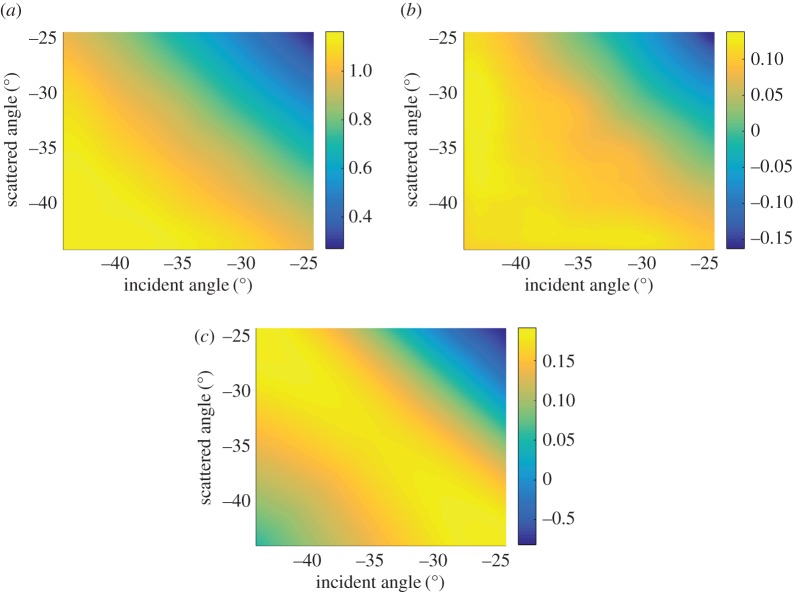
Scattering matrix of a surface-breaking crack with parameters *a*_crack_= 1.13λ, *θ*_crack_=45°: (*a*) measured scattering matrix; (*b*) noise part of the scattering matrix; (*c*) one realization of Gaussian coherent noise with parameters *σ*_coh_=0.1, λ_1coh_=30°, λ_2coh_=10° and *ϕ*_coh_=−45°. (Online version in colour.)

Figure 6*a* shows the experimentally measured absolute value of the scattering matrix for a surface-breaking crack of 1.13λ length and 45° orientation. The experimental set-up is the same as described in §2 (figure 1). The difference between the measured scattering matrix and the simulated scattering matrix for the crack with the same parameters (noise part of the scattering matrix) is shown in figure 6*b*. It can be seen that the noise is coherent, different components of the noise scattering matrix are not independent and are seen to be correlated with each other. Moreover, in this particular case the structure of the noise scattering matrix is very similar to the structure of the scattering matrix itself. Note that here we do not specifically comment on the source of the noise and attempt to develop a general model.

The noise part of the scattering matrix can be considered as a rough two-dimensional surface and, hence, various models of surface roughness can be applied to model the coherent noise. In this case, the amplitude of each component of the noise scattering matrix is described by some probability density function, and the correlation between different components is described by a correlation function. In this paper, a Gaussian model of correlated noise is chosen.

Note that generally the noise affects both the amplitude and phase of the scattering matrix, or its real and imaginary parts. The experimental complex-valued scattering matrix *S*_exp_(*θ*_in_,*θ*_sc_) can be represented in the form

5.1 $$S_{\exp}=S_{p}+S_{\mathrm{coh},1}+iS_{\mathrm{coh},2},$$

where *S*_*p*_ is the noiseless scattering matrix. The real functions *S*_coh,*n*_, *n*=1,2, are now statistically described by the same probability density function *ρ*_coh_(*S*). For simplicity, it is assumed that the mean value of each component of the noise scattering matrix is zero. This also implies that the average value of the noise scattering matrix is zero, so

5.2 $$\langle S_{\mathrm{coh},n}\rangle =0,\;n=1,2,$$

where the angled brackets denote averaging over incident and scattered angles.

The correlation function represents the extent to which the noise at one point (*θ*_in_,*θ*_sc_) determines the noise at some point (*ζ*_1_,*ζ*_2_) away, and is defined as

5.3 $$C(\zeta_{1},\zeta_{2})=\frac{1}{\sigma_{\mathrm{coh}}^{2}}\langle S_{\mathrm{coh},n}(\theta_{\mathrm{in}},\theta_{\mathrm{sc}})S_{\mathrm{coh},n}(\theta_{\mathrm{in}}+\zeta_{1},\theta_{\mathrm{sc}}+\zeta_{2})\rangle .$$

To simplify analytical manipulations, both the noise amplitude distribution and correlation functions are regarded as Gaussian. In this case, the probability density function $\rho_{\mathrm{coh}}∼N(0,\sigma_{\mathrm{coh}}^{2})$, where *σ*_coh_ is the standard deviation of noise amplitude. The two-dimensional correlation function *C*(*ζ*_1_,*ζ*_2_) can be written in the form

5.4 $$C(\zeta_{1},\zeta_{2})=\exp\left(-\frac{{\tilde{\zeta }}_{1}^{2}}{\lambda_{1\mathrm{coh}}^{2}}-\frac{{\tilde{\zeta }}_{2}^{2}}{\lambda_{2\mathrm{coh}}^{2}}\right)$$

and

5.5 $$\left(\begin{array}{c} {\tilde{\zeta }}_{1}\\ {\tilde{\zeta }}_{2} \end{array}\right)=\text{R}\left(\begin{array}{c} \zeta_{1}\\ \zeta_{2} \end{array}\right),\;\text{R}=\left(\begin{array}{cc} \cos\phi_{\mathrm{coh}} & \sin\phi_{\mathrm{coh}}\\ -\sin\phi_{\mathrm{coh}} & \cos\phi_{\mathrm{coh}} \end{array}\right).$$

Here, the isolines of the correlation function represent ellipses with the axes λ_1coh_,λ_2coh_, rotated by the angle *ϕ*_coh_with respect to the coordinate system (*ζ*_1_,*ζ*_2_).

A particular realization of a coherent noise can be simulated by the convolution of the correlation function, with uncorrelated unit variance and zero mean Gaussian white noise *n*_*g*_(*θ*_in_,*θ*_sc_)

5.6 $$S_{\mathrm{coh},n}(\theta_{\mathrm{in}},\theta_{\mathrm{sc}})=(C(\theta_{\mathrm{in}},\theta_{\mathrm{sc}})⊗n_{g}(\theta_{\mathrm{in}},\theta_{\mathrm{sc}})-\mu^{\prime })\frac{\sigma_{\mathrm{coh}}}{\sigma^{\prime }},$$

where ⊗ is the convolution operator, and *μ*′ and *σ*′ are, respectively, the mean and standard deviations of the convolution *C*⊗*n*_*g*_ over incident and scattered angles [37].

In this paper, only an amplitude of the scattering matrix, |*S*_exp_|, is considered. If the noise amplitude is small, *σ*_coh_≪|*S*_*p*_|, then the amplitude of the scattering matrix can be approximately written as

5.7 $$|S_{\exp}|=|S_{p}|+S_{\mathrm{coh}},\;S_{\mathrm{coh}}=\frac{ℜ[S_{p}]}{|S_{p}|}S_{1\mathrm{coh}}+\frac{ℑ[S_{p}]}{|S_{p}|}S_{2\mathrm{coh}}.$$

Because the sum of two independent normally distributed variables also has a normal distribution, then from (5.7) it follows that *S*_coh_ is also described by the model (5.6).

Figure 6*c* shows one realization of the coherent noise for the parameters *σ*_coh_=0.1, λ_1coh_=30°, λ_2coh_=10° and *ϕ*_coh_=−45°. It can be seen that the structure of the simulated noise scattering matrix is very similar to the experimental noise scattering matrix shown in figure 6*b*.

In pc-space, each scattering matrix is represented by an *N*_*s*_-dimensional vector, so expression (5.7) can be written in a form similar to expression (4.1)

5.8 $${\text{s}}_{\exp}^{(\mathrm{pc})}={\text{s}}_{p}^{\left(\mathrm{pc}\right)}+{\text{n}}_{\mathrm{coh}}^{\left(\mathrm{pc}\right)},$$

where the noise vector ${\text{n}}_{\mathrm{coh}}^{\left(\mathrm{pc}\right)}$ corresponds to the noise scattering matrix *S*_coh_. Below, it is assumed for simplicity that the noise is independent of the defect parameter point and described by the same parameters *σ*_coh_, λ_1coh_, λ_2coh_ and *ϕ*_coh_ for all points on the *d*-manifold.

The defect characterization method requires the knowledge of the probability density function *ρ*_*S*_(**s**^(pc)^_exp_ | **p**). Using expression (5.8) and taking into account that the noise does not depend on the defect parameter **p**, the function *ρ*_*S*_ can be written as

5.9 $$\rho_{S}({\text{s}}_{\exp}^{(\mathrm{pc})}\;|\;\text{p})=\rho_{S}({\text{s}}_{p}^{\left(\mathrm{pc}\right)}+{\text{n}}_{\mathrm{coh}}^{\left(\mathrm{pc}\right)}\;|\;\text{p})=\rho_{n}({\text{n}}_{\mathrm{coh}}^{\left(\mathrm{pc}\right)}),$$

where *ρ*_*n*_ is the probability density function of the noise vector **n**^(pc)^_coh_. An important advantage of the proposed coherent noise model is that the function *ρ*_*n*_(**n**^(pc)^_coh_) can be written in an explicit form and the details are given in appendix Ab. Moreover, as shown in appendix Ab, the characterization result (defect parameters corresponding to the highest probability) can be found by the projection of the measurement point on the *d*-manifold in the normalized noise pc-space, which represents the rotated and scaled defect class pc-space. Or, alternatively, this projection can be performed in defect class pc-space, but using a different metric, which corresponds to the distance in the normalized noise pc-space.

**Figure 7.**
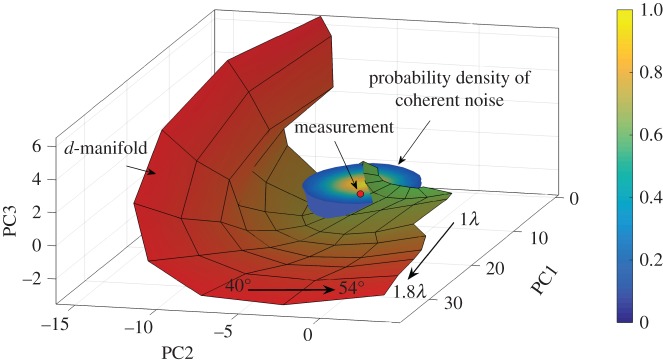
Probability density function (normalized to its maximum value) of Gaussian coherent noise *ρ*_*S*_(**s**^(pc)^ | **p**) in the pc-space of surface-breaking cracks at the crack’s parameter point *a*_crack_=1.13λ, *θ*_crack_=45°. Noise parameters are *σ*_coh_=0.1, λ_coh,1_=30°, λ_coh,2_=10° and *ϕ*_coh_=−45°. Colourmap of the manifold is given by figure 4*a*; colourbar is related to the probability density function.

To illustrate the distribution of noise in pc-space the *d*-manifold for surface-breaking cracks is considered. The experimental set-up is assumed to be the same as in §2 (figure 1). The noise probability density function was calculated using expression (8.10) with noise parameters *σ*_coh_=0.1, λ_coh,1_=30°, λ_coh,2_=10° and *ϕ*_coh_=−45°. Figure 7 shows the noise distribution around the point on the *d*-manifold corresponding to the crack parameters *a*_crack_=1.13λ and *θ*_crack_=45°. It can be seen that the noise distribution in the defect class pc-space has the shape of an ellipsoid which is rotated relative to the defect class principal components coordinate system. Then, the position of the measurement point **s**^(pc)^_exp_ inside this ellipsoid defines the probability density function *ρ*_*S*_(**s**^(pc)^_exp_ | **p**), **p**={*a*_crack_,*θ*_crack_}, and hence the probability that the measurement corresponds to a particular set of defect parameters.

Note that the Bayesian statistical approach described in §4 is similar to the method previously used in [15] for characterization of corrosion defects in plates. However, the method proposed in this paper has several major differences. Firstly, the characterization is performed in pc-space, which provides a simple geometrical interpretation of the characterization process and also allows us to filter random experimental noise from the measurements [12]. Secondly, the fact that the characterization uncertainty defined by the noise model is highlighted and a general model of the coherent noise is developed.

## 6. Experiments

### (a) Experimental characterization procedure

**Figure 8.**
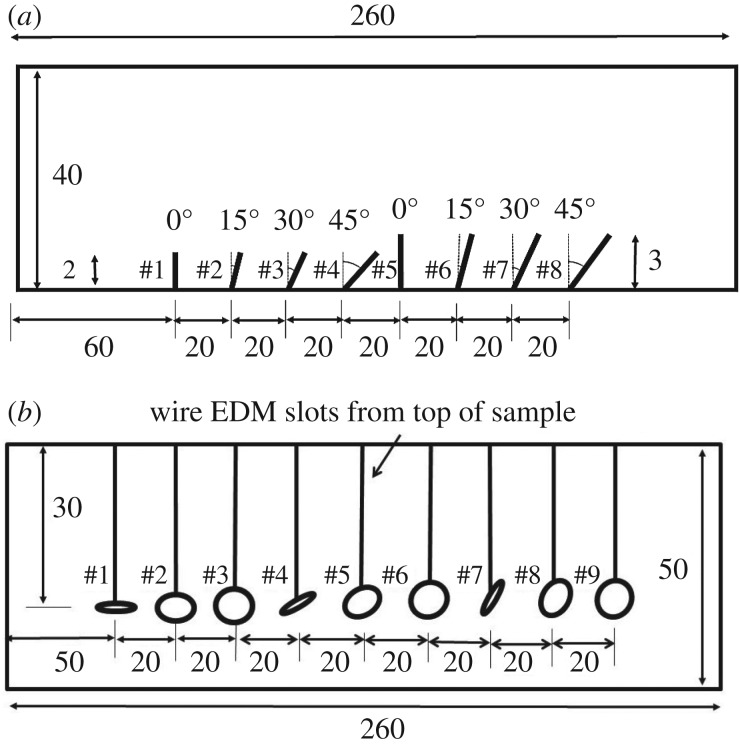
(Dimensions are in millimeters.) Experimental sample geometries containing: (*a*) electrical discharge machining (EDM) notches; (*b*) elliptical voids.

Experimentally, the performance of the characterization method is studied on two different defect types: surface-breaking cracks and elliptical voids (see figure 8 for the sample geometries). As discussed earlier, both defects are of particular industrial interest, and they have different numbers of parameters of interest (two for surface-breaking cracks and three for elliptical voids). The true defect parameters are given in tables 1 and 2. In both cases, the *s*-spaces include the scattering matrices of defects with sizes between 0.5λ and 2λ, as the scattering matrix is most informative within this size range [11]. For defects larger than 2λ, their characterization should be possible directly from high-resolution ultrasonic images, such as the ones obtained with the total focusing method (TFM) [38]. The considered angle range of surface-breaking cracks is from −60° to 60°. For elliptical voids, aspect ratios between 0.1 and 0.9 and ellipse orientation angles between −90° and 90° are considered. Note that the orientation angle of elliptical voids is measured with respect to the array direction, and the angle of a horizontal ellipse is 0°. Therefore, orientation angle of the crack, *θ*_crack_, is related to the orientation angle of the void, *θ*_void_, by

6.1 $$\theta_{\mathrm{crack}}+\theta_{\mathrm{void}}=90^{\circ }.$$

**Table 1.** Parameters of the machined notches shown in figure 8*a*.

	parameters	
defect	size (λ)	angle (°)
1	0.80	0
2	0.83	15
3	0.92	30
4	1.13	45
5	1.20	0
6	1.24	15
7	1.39	30
8	1.70	45

**Table 2.** Parameters of the elliptical voids shown in figure 8*b*. AR represents the aspect ratio.

	parameters		
defect	AR	size (λ)	angle (°)
1	0.32	1.00	0
2	0.50	1.00	0
3	0.80	1.00	0
4	0.32	1.00	30
5	0.50	1.00	30
6	0.80	1.00	30
7	0.32	1.00	60
8	0.50	1.00	60
9	0.80	1.00	60

In this paper, the sub-array imaging technique [39] is used to extract the scattering matrices from experimental data. In this case, the amplitude of the scattering matrix also needs to be normalized by comparing with a reference scatterer. Here, the back wall of the specimen was used as a reference scatterer. The corresponding array data can be simulated using the hybrid model introduced in [40]. To extract the absolute amplitude of the scattering matrix correctly, the experimental array data are multiplied by a constant gain, which makes the maximum TFM image amplitude of the reference scatterer for experimental and simulated data equal.

Note that the sub-array imaging technique introduces a specific coherent noise to the extracted scattering matrices. In particular, the value of the scattering matrix at any incident and scattered angles is represented by an averaging of the array data from several consecutive array elements (i.e. sub-array). This results in a ‘smoothing’ effect relative to the true scattering matrix [39]. Since this is a deterministic effect, it can be resolved by constructing a ‘sub-array version’ of the scattering matrix in *s*-space. The sub-array scattering matrix, *S*_sa_, can be obtained by

6.2 $$S_{\mathrm{sa}}(\theta_{\mathrm{in},k}^{a},\theta_{\mathrm{sc},l}^{a})=\frac{1}{N_{\mathrm{sa}}^{2}}\sum_{i\in a_{k},j\in a_{l}}S(\theta_{\mathrm{in},i},\theta_{\mathrm{sc},j}),$$

where *a*_*k*_ denotes the *k*th sub-array aperture with the corresponding incident, $\theta_{\mathrm{in},k}^{a}$, and scattered, $\theta_{\mathrm{sc},k}^{a}$, angles. The value of the scattering matrix *S*(*θ*_in,*i*_,*θ*_sc,*j*_) corresponds to the *i*th transmitter element and the *j*th receiver element in the full array aperture.

To calculate the probability density function *ρ*_*p*_(**p** | **s**^(pc)^_exp_) of defect parameters the Gaussian coherent noise model developed in §5 was used. The noise parameters *σ*_coh_, λ_coh,1_, λ_coh,2_ and *ϕ*_coh_ were defined from comparison of the measured scattering matrices with modelled scattering matrices of the defects with ideal geometries using the maximum-likelihood estimation method. In this case, expression (8.8) for the noise probability density function in the defect class pc-space was used. For the case of surface-breaking cracks, the maximum of the likelihood function is achieved for the noise parameters *σ*_coh_=0.1, λ_coh,1_=30°, λ_coh,2_=10°, *ϕ*_coh_=45°. For the case of elliptical voids, the noise parameters were estimated as *σ*_coh_=0.1, λ_coh,1_=20°, λ_coh,2_=10°, *ϕ*_coh_=45°.

For each defect the probability density map corresponding to each defect class (surface-breaking cracks and elliptical voids) was calculated using expressions (4.3), (4.4), (5.9) and (8.8). Note that if the *d*-manifold for the defect class is defined by some sampling scheme, then any point in the parameter space can be mapped into the principal components pc-space. Practically, this means that, although the sampling of the *d*-manifold is finite, the probability density map in the parameter space can be calculated with any resolution.

It should be stressed that the developed defect characterization approach does not give just a single answer. The method provides quantitative information about possible defect parameters and, more importantly, about the confidence level of the characterization result. The main output of the characterization procedure is the probability density map of the defect parameters, *ρ*_*p*_(**p** | **s**^(pc)^_exp_). The characterization result is then given by the most probable parameters, **p**_*c*_, where the probability density function has the maximum value.

The structure of the probability function in the defect parameter space can be characterized by the square root of the second moment of the probability distribution about the characterization point **p**_*c*_,

6.3 $$\sigma_{i}^{\left(p\right)}=\sqrt{\int {\left(p_{i}-p_{c,i}\right)}^{2}\rho_{p}(\text{p}\;|\;{\text{s}}_{\exp}^{(\mathrm{pc})})\;dp},$$

where the index *i* denotes the *i*th component of the vectors **p** and **p**_*c*_, and the integral is calculated over all parameter space. The physical meaning of the value $\sigma_{i}^{\left(p\right)}$ is a standard deviation of the *i*th defect parameter about the characterization point.

The relative noise amplitude, or the relative error of the characterization result, can be defined as

6.4 $${\tilde{a}}_{\mathrm{noise}}=\frac{|{\text{s}}_{\exp}^{\left(\mathrm{pc}\right)}-{\text{s}}_{c}^{\left(\mathrm{pc}\right)}|}{|{\text{s}}_{c}^{\left(\mathrm{pc}\right)}|},$$

where the vector ${\text{s}}_{c}^{\left(\mathrm{pc}\right)}$ corresponds to the characterization point on the *d*-manifold in the pc-space. Note that the relative noise amplitude can also be used to compare the characterization results in two defect classes.

Another important characteristic of the uncertainty of the characterization result is the *d*-index of the defect class at the characterization point. The *d*-index is defined by expression (3.9) and describes the sensitivity of the characterization result to the noise. For each defect class, the *d*-index characterizes the structure of the defect class *d*-manifold and depends on the amount of information in the measured part of the scattering matrix. Application of these parameters for interpretation of the characterization results is illustrated in the next section.

### (b) Results for surface-breaking cracks

The aluminium test specimen shown in figure 8*a* contains eight EDM notches. The vertical depth of defects 1–4 is 2 mm, and defects 5–8 have the same vertical depth of 3 mm. The actual size of the defects can be calculated as *d*/*cos*(*α*), where *d* and *α* represent the vertical depth and the orientation angle, respectively. As a result, the studied defects have sizes between 0.8λ (defect 1) and 1.7λ (defect 8), and angles between 0° and 45°. The array measurement configuration is shown in figure 1. Note that the array is moved away from the crack by 30 mm, because otherwise the crack would be obscured by the high-intensity reflection from the back wall in the image. Therefore, the scattering matrix for each crack was measured for the −45°≤*θ*_in_,*θ*_sc_≤−23° angular interval.

**Figure 9.**
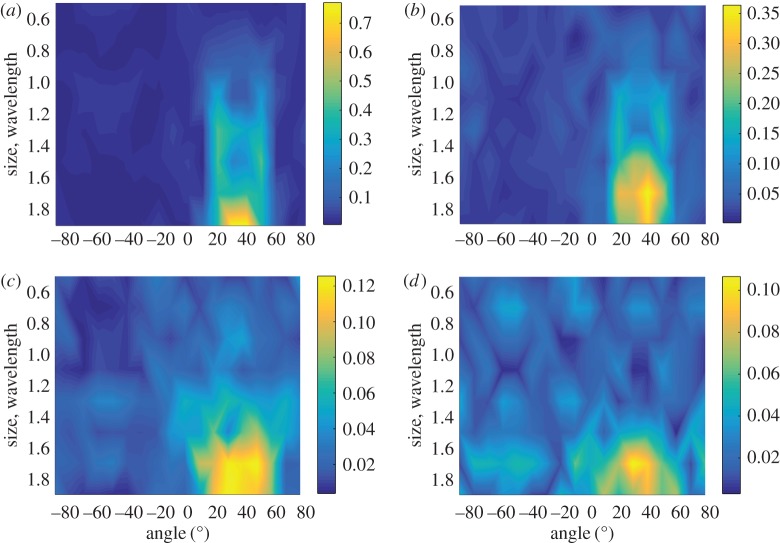
Map of the *d*-index for the elliptical voids corresponding to the −45°≤ *θ*_in_,*θ*_sc_≤−23° part of the scattering matrix. Each figure represents a slice at constant aspect ratio equal to (*a*) 0.1, (*b*) 0.3, (*c*) 0.6, (*d*) 0.9. (Online version in colour.)

The *d*-indexes for the surface-breaking cracks and elliptical voids corresponding to the measurement angular interval are shown in figures 5*b* and 9, respectively. Taking into account the relationship (6.1) between crack and void orientation angles, it can be seen that, in both cases, the most stable parameter regions correspond to the similar defect geometries: 1λ≤*a*_crack_≤2λ, 40°≤*θ*_crack_≤60° for the cracks and 1λ≤*a*_void_≤2λ, 30°≤*θ*_void_≤50° for narrow elliptical voids with aspect ratio between 0.1 and 0.3. Note that for these defect orientation angles the measured part of the scattering matrix contains the specular reflection and, therefore, it seems reasonable that this is the most informative part from the defect characterization point of view.

**Figure 10.**
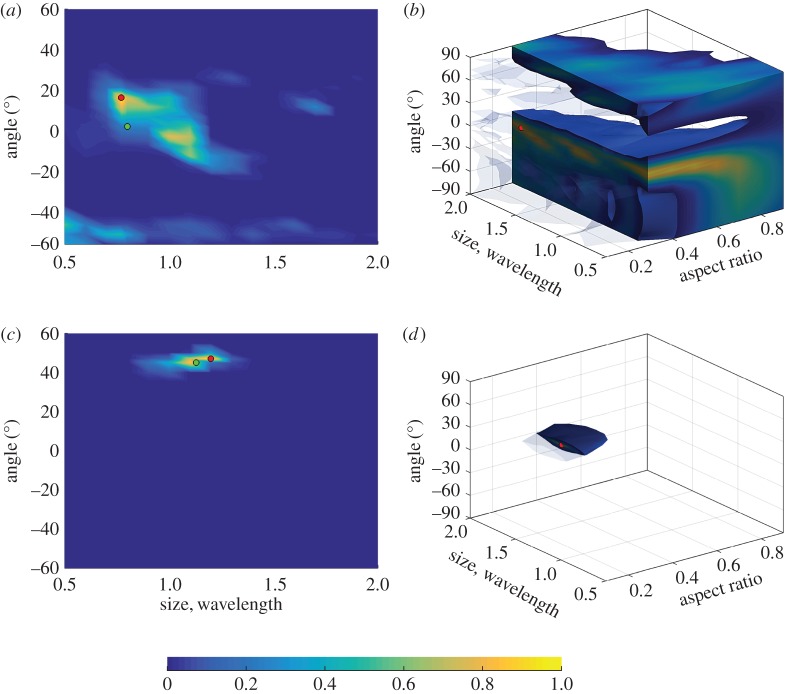
Probability maps of the machined notches shown in figure 8*a* for defects 1 (*a*,*b*) and 4 (*c*,*d*), characterized in the surface-breaking crack *s*-space (*a*,*c*) and in the void *s*-space (*b*,*d*). The red dots indicate the maximum probability point; the green dots are the true parameter values. The probabilities shown here are normalized to the maximum value.

The characterization results of the machined notches in the surface-breaking crack defect class are summarized in table 3. The corresponding probability density functions of defect parameters are illustrated in figure 10 for defects 1 and 4.

**Table 3.** Characterization results of the machined notches shown in figure 8*a* in the surface-breaking crack defect class. $\sigma_{l}^{\left(p\right)}$ and $\sigma_{\theta }^{\left(p\right)}$ correspond to the standard deviation of defect size and angle about the characterization point.

					characterization results, cracks in crack database	
defect	size (λ)	angle (°)	*d*-index	$\sigma_{l}^{\left(p\right)}$ (λ)	$\sigma_{\theta }^{\left(p\right)}$ (°)	${\tilde{a}}_{\mathrm{noise}}$
1	0.77	14.88	0.024	0.32	33.92	0.033
2	1.10	−15.00	0.035	0.28	28.75	0.080
3	0.97	27.87	0.059	0.35	46.96	0.078
4	1.21	47.03	0.150	0.14	2.45	0.063
5	1.31	15.00	0.033	0.27	19.30	0.480
6	1.79	10.00	0.035	0.82	21.44	0.250
7	1.44	30.33	0.040	0.04	1.40	0.090
8	1.70	46.71	0.230	0.06	0.11	0.140

Firstly, it is noted that, for all defects, the correct defect parameters are located in the range of the probability density function (see figure 10 as an example). Therefore, true parameters represent a possible characterization point in the parameter space, although this point does not always correspond to the highest probability.

From table 3, it can be seen that the standard deviations of defect parameters about the characterization point are well correlated with the values of the *d*-index. This confirms that the confidence level of the characterization results is defined by the global structure of the *d*-manifold. According to the discussion on the *d*-index, defects 4 and 8 are favourably oriented, i.e. have high *d*-index values. Consequently, the characterization results show that these two defects are correctly characterized with high confidence (figure 10*c* for defect 4). For the defects 1–3, 5 and 6, the *d*-indexes are low. This is in agreement with probability maps (figure 10*a* for defect 1), which show that, for these defects, there exist multiple regions of high probability in the defect parameter spaces and, therefore, the characterization uncertainty is large.

Now, the characterization of crack defects in the elliptical void defect class is considered. The corresponding results are given in table 4. The probability density in this case is a function of three parameters and is shown in figure 10*b*,*d* for defects 1 and 4. The characterization results for different defect classes can be compared using the relative noise amplitudes of the characterization results, ${\tilde{a}}_{\mathrm{noise}}$, and the characterization defect class is defined as the class with the minimum value of ${\tilde{a}}_{\mathrm{noise}}$. Note that ${\tilde{a}}_{\mathrm{noise}}=0$ for defects 1–3. Geometrically, it means that the corresponding experimental point **s**^(pc)^_exp_ is exactly located on the *d*-manifold.

**Table 4.** Characterization results of the machined notches shown in figure 8*a* in the void defect class. AR represents the aspect ratio; $\sigma_{\mathrm{AR}}^{\left(p\right)}$, $\sigma_{l}^{\left(p\right)}$ and $\sigma_{\theta }^{\left(p\right)}$ correspond to the standard deviation of the defect aspect ratio, size and angle about the characterization point, respectively.

							characterization results, cracks in void database	
defect	AR	size (λ)	angle (°)	*d*-index	$\sigma_{\mathrm{AR}}^{\left(p\right)}$	$\sigma_{l}^{\left(p\right)}$ (λ)	$\sigma_{\theta }^{\left(p\right)}$ (°)	${\tilde{a}}_{\mathrm{noise}}$
1	0.29	1.89	−11.72	0.013	0.260	0.88	45.66	0
2	0.25	1.10	5.68	0.010	0.260	0.43	56.29	0
3	0.21	1.25	56.96	0.140	0.210	0.39	45.58	0
4	0.19	1.21	42.68	0.180	0.070	0.13	1.76	0.03
5	0.11	1.85	53.46	0.230	0.460	0.72	66.00	0.39
6	0.16	1.77	55.83	0.210	0.490	0.73	79.47	0.40
7	0.27	1.08	7.29	0.014	0.260	0.43	50.34	0.44
8	0.10	1.86	43.13	0.610	0.009	0.07	0.14	0.09

From table 4, it can be seen that only defects 6 and 7 are classified as surface-breaking cracks. According to the minimum relative noise amplitude criterion, all other defects should be classified as voids. However, it can be seen that, in the elliptical void class, all surface-breaking cracks are characterized as narrow ellipses with small aspect ratio (e.g. 0.2). Also, the results for defects 1–3, 5 and 6 have very large uncertainty in both defect classes, confirming that, for these defects, the amount of information in the measured part of the scattering matrix is not enough for unique defect characterization.

On the other hand, the characterization results of defects 4 and 8 in the void class have similar sizes and orientation angles to the true crack parameters. So, in this case, the characterization result in the void defect class brings confidence, confirming that the defects are crack-like and the method is providing correct defect parameters. Physically, this means that, for these defects, the influence of the back wall on the scattering behaviour in the considered angular measurement range is small. Consequently, the measured parts of the scattering matrices for surface-breaking cracks and isolated cracks are very similar to each other. Note that, in practice, a more sophisticated model of defect class prior probability *ρ*_*p*_(**p**) can be adopted based on specific measurement scenarios. For example, if a defect is found near the surface, then it is more likely to be a surface-breaking crack than a void.

### (c) Results for elliptical voids

The array measurements of the specimen containing elliptical voids were performed by positioning the array exactly above each defect. For this experimental configuration the measured part of the scattering matrix corresponds to the angles −30°≤*θ*_in_,*θ*_sc_≤30°. As in the previous section, all defects were characterized in the two defect classes: elliptical voids and surface-breaking cracks. However, for all elliptical defects the relative noise amplitude was smaller for the characterization results corresponding to the void defect class. Therefore, all defects were characterized as voids, and, below, the results only for the void defect class are discussed.

**Figure 11.**
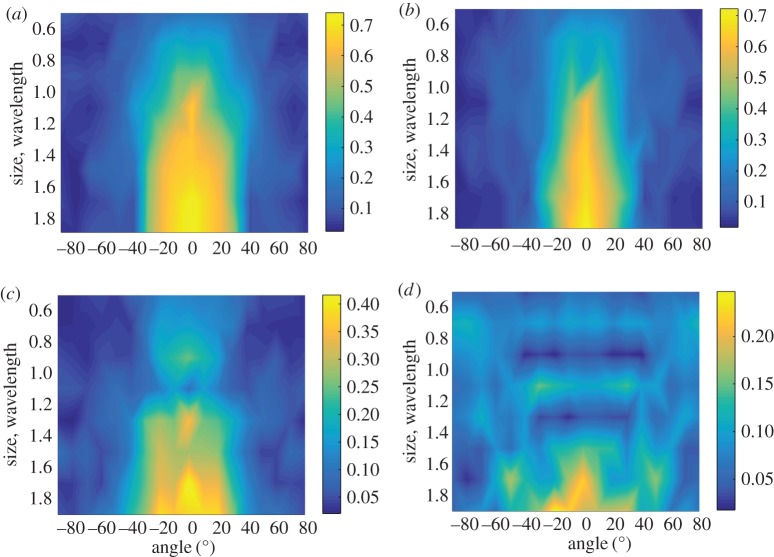
Map of the *d*-index for the elliptical voids corresponding to the −30°≤ *θ*_in_,*θ*_sc_≤30° part of the scattering matrix. Each figure represents a slice at constant aspect ratio equal to (*a*) 0.1, (*b*) 0.3, (*c*) 0.6, (*d*) 0.9. (Online version in colour.)

The *d*-index for the elliptical void defect class is shown in figure 11. From this figure, it follows that the defect parameters can be estimated with relatively high confidence for ellipses with aspect ratios between 0.1 and 0.6, sizes between 1λ and 2λ, and orientation angles between −30° and 30°. Note that, for these defect orientation angles, the measured part of the scattering matrix contains specular reflection.

**Figure 12.**
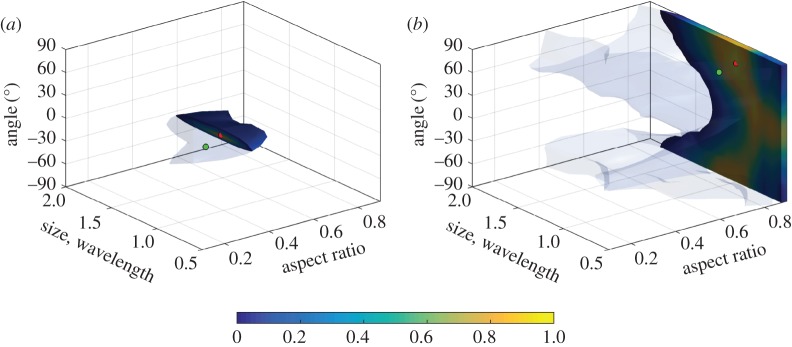
Probability maps of the elliptical voids 1 (*a*) and 9 (*b*) (figure 8*b*) characterized in the void *s*-space. The slice through the three-dimensional probability map at the constant aspect ratio, corresponding to the maximum probability point, is shown. The red dots indicate the maximum probability point and the green dots are the true parameter values. The probabilities shown here are normalized to the maximum value.

The characterization results of the elliptical voids are summarized in table 5 and the probability density functions for defects 1 and 9 are shown in figure 12. Note that, similar to the surface-breaking crack characterization, all true defect parameters are located in the range of the corresponding probability density functions in the defect parameter space. It is seen that the confidence level of the characterization is correlated with the values of the *d*-index. For example, the characterization result of defect 1 has the highest *d*-index, and this defect is correctly characterized with high confidence. The corresponding probability density function in figure 12*a* is focused around the true parameter point in the defect parameter space.

**Table 5.** Characterization results of the elliptical voids shown in figure 8*b* in the void defect class. AR represents the aspect ratio; $\sigma_{\mathrm{AR}}^{\left(p\right)}$, $\sigma_{l}^{\left(p\right)}$ and $\sigma_{\theta }^{\left(p\right)}$ correspond to the standard deviation of the defect aspect ratio, size and angle about the characterization point, respectively.

						characterization results, voids in void database		
defect	AR	size (λ)	angle (°)	*d*-index	$\sigma_{\mathrm{AR}}^{\left(p\right)}$	$\sigma_{l}^{\left(p\right)}$ (λ)	$\sigma_{\theta }^{\left(p\right)}$ (^°^)	${\tilde{a}}_{\mathrm{noise}}$
1	0.43	1.09	1.56	0.53	0.08	0.20	2.75	0.03
2	0.51	1.11	4.00	0.38	0.17	0.38	13.18	0.06
3	0.77	1.17	8.00	0.12	0.10	0.40	41.23	0.02
4	0.34	1.80	36.81	0.16	0.12	0.64	8.26	0.21
5	0.54	1.05	24.83	0.12	0.15	0.47	21.83	0.15
6	0.78	0.97	14.38	0.06	0.10	0.51	46.94	0.02
7	0.42	0.95	60.65	0.09	0.13	0.46	29.17	0.02
8	0.61	0.72	51.47	0.04	0.15	0.56	30.74	0.06
9	0.87	0.99	66.00	0.08	0.17	0.44	42.27	0.25

On the other hand, the value of the *d*-index for defect 9 is very low, which results in the high characterization uncertainty seen in figure 12*b*. Note that the scattering matrix of voids with an aspect ratio close to 1 is insensitive to defect orientation angle, so low values of the *d*-index in this case correspond to uncertainty with respect to the orientation angle. It is also seen that, although the defect size is correctly characterized, its uncertainty is also large. However, the aspect ratio is estimated correctly and the corresponding standard deviation is small.

## 7. Conclusion

A new defect characterization approach which can potentially be applied to any defect databases (on condition that the defects are parameterized with several continuous variables) has been proposed in this paper. In this method, the defect database has the form of a parametric manifold, and can be approximated to any resolution from a finite number of samples. The characterization problem can be formulated as calculating the posterior probability *ρ*_*p*_(**p** | *S*_exp_) for every possible parameter value **p** given the occurrence of the measured scattering matrix *S*_exp_. The result is dependent on the specific noise model which is described by a probability density function *ρ*_*S*_(*S*_exp_ | **p**) of the measured scattering matrix given the occurrence of the defect with parameters **p**. A general Gaussian coherent noise model is proposed, and the analytical expression for the corresponding probability density function is derived.

The defect characterization data provided by the developed method can be represented in different forms and described by different parameters. For example, the probability distribution in the defect parameter space can be quantified by the standard deviation of defect parameters about the characterization point (see expression (6.3)). The sensitivity of the characterization result to noise can be described by the value of the *d*-index, which captures the structure of the *d*-manifold. To assess the general confidence of the characterization result and compare results obtained in the different defect classes, the relative noise amplitude (expression (6.4)) can be used, which describes how closely the experimental *S*-matrix matches the characterization result. Moreover, the developed defect characterization method allows us to define other quantitative characterization parameters, which can be useful for specific practical applications.

Experimentally, the proposed approach is used to characterize surface-breaking defects and elliptical voids. Note that one of the advantages of the proposed method is its ability to quantify the defect characterization uncertainty. In general, the characterization results are accurate, except for the cases where the defects are unfavourably oriented, which results in a lack of useful information in the array data. It is also concluded that the characterization results of unfavourably oriented defects tend to have higher uncertainty. Furthermore, reliable estimation of the noise parameters are required for accurate quantification of the characterization uncertainty.

## Supplementary Material

Experimentally measured scattering matrices of the machined notches.

## Supplementary Material

Experimentally measured scattering matrices of the elliptical defects.

## Data accessibility

The datasets supporting this article have been uploaded as part of the electronic supplementary material.

## A. Appendix

**(a) Sampling of defect manifold**

In this section, the problem of practical implementation of the characterization procedure proposed in §4 is considered. In general, the defect *d*-manifold cannot be described analytically and, therefore, has to be defined by some sampling. Firstly, the parameter space is sampled and then triangulated using, for example, Delaunay triangulation [41,42]. Then, for any parameter vector **p**, the position of the point *S*_*p*_(**p**) on the *d*-manifold can be approximately found by using an interpolation scheme and, hence, the *d*-manifold can be approximated with any resolution. Inversely, any point on the *d*-manifold can be approximately mapped back into the parameter space. Therefore, the structure of the *d*-manifold is completely defined by its sampling points.

In pc-space, each *N*_*p*_-dimensional triangle is given by *N*_*p*_+1 sampling points **s**^(pc)^_*n*_, *n*=0…,*N*_*p*_ and defines a ‘facet’ of the *d*-manifold. The local coordinate system in each ‘facet’ is described by the *N*_*p*_ vectors ${\text{w}}_{n}={\text{s}}_{n}^{(\mathrm{pc})}-{\text{s}}_{0}^{\left(\mathrm{pc}\right)},\;n=1,\ldots ,N_{p}$. Then, any scattering matrix, **s**^(pc)^, as a vector in pc-space can be represented in the form

A 1 $${\text{s}}^{\left(\mathrm{pc}\right)}={\text{s}}_{0}^{\left(\mathrm{pc}\right)}+{\text{s}}_{∥}^{\left(\mathrm{pc}\right)}+{\text{s}}_{⊥}^{\left(\mathrm{pc}\right)},\;{\text{s}}_{∥}^{\left(\mathrm{pc}\right)}=\sum_{n=1}^{N_{p}}b_{n}{\text{w}}_{n}.$$

Here, the vector **s**^(pc)^_∥_ represents the projection of the vector **s**^(pc)^ onto the facet subspace and the vector **s**^(pc)^_⊥_ is orthogonal to the facet subspace. Therefore, the distance from the point **s**^(pc)^ to the facet subspace is equal to $|{\text{s}}_{⊥}^{(\mathrm{pc})}|=|{\text{s}}^{\left(\mathrm{pc}\right)}-{\text{s}}_{∥}^{\left(\mathrm{pc}\right)}-{\text{s}}_{0}^{\left(\mathrm{pc}\right)}|$. The coefficients *b*_*n*_ are the coordinates of the vector **s**^(pc)^_∥_ in the facet local coordinate system. Based on (A 1), it can be shown that

A 2 $$\text{b}=\text{B}({\text{s}}^{\left(\mathrm{pc}\right)}-{\text{s}}_{0}^{\left(\mathrm{pc}\right)}),\;\text{B}={\text{G}}^{-1}{\text{W}}^{T},$$

where **b**={*b*_1_,…,*b*_*N*_*p*__}^*T*^, and the matrices **G**,**V** are given by *G*_*ij*_=(**w**_*i*_,**w**_*j*_), *W*_*ij*_=*w*_*i*,*j*_.

In §4, it has been shown that if the noise is isotropic in pc-space, then the characterization point is given by the projection of the measured scattering matrix **s**^(pc)^_exp_ onto the *d*-manifold. Expression (A 1) shows that the problem of finding the projection is equivalent to the following optimization problem:

A 3 $$\min_{\text{all facets}}|{\text{s}}_{\exp}^{(\mathrm{pc})}-{\text{s}}_{\exp,∥}^{\left(\mathrm{pc}\right)}-{\text{s}}_{0}^{\left(\mathrm{pc}\right)}|,\;b_{n}\geq 0,\;\sum_{n=1}^{N_{p}}b_{n}\leq 1,$$

where ${\text{s}}_{\exp}^{\left(\mathrm{pc}\right)}$ is the vector in the pc-space corresponding to the scattering matrix *S*_exp_, the coefficients *b*_*n*_ are calculated from (A 2) and the minimum is taken over all facets. In some cases, it is possible that the problem (A 3) does not have a solution. Then, the point on the *d*-manifold corresponding to the shortest distance is located on the boundary of some facet. In this case, the minimization procedure (A 3) can be iteratively applied to the boundary of each facet.

Once the projection point is found, it can be mapped back into the parameter space. If the projection facet is defined by the points **s**^(pc)^_*n*_, *n*=0,…,*N*_*p*_, which correspond to the parameter vectors **p**_*n*_, ${\text{s}}_{n}^{(\mathrm{pc})}={\text{s}}_{p}^{\left(\mathrm{pc}\right)}({\text{p}}_{n})$, then the characterization parameter vector, **p**_*c*_, is given by

A 4 $${\text{p}}_{c}={\text{p}}_{0}+\sum_{n=1}^{N_{p}}b_{n}({\text{p}}_{n}-{\text{p}}_{0}).$$

**(b) Probability density function of coherent noise**

In this section, it is shown that the probability density function of the Gaussian coherent noise, *ρ*_*n*_(**n**^(pc)^_coh_), can be written in the analytical form. The first step is to apply the PCA to the set of noise vectors **n**^(pc)^_coh_. The noise vector in the new noise pc-space will be denoted as **n**^(*pc_n_*)^_coh_, where the *pc*_*n*_ index specifically indicates that the noise pc-space is different from the defect class pc-space. Mathematically, the transformation of **n**^(pc)^_coh_ into ${\text{n}}_{\mathrm{coh}}^{\left({\mathrm{pc}}_{n}\right)}$ is the same as described in §3a (equations (3.3)–(3.5)). Note that the mean of **n**^(pc)^_coh_ is zero, therefore the transformation of ${\text{n}}_{\mathrm{coh}}^{\left(\mathrm{pc}\right)}$ into **n**^(*pc_n_*)^_coh_ is equivalent to the rotation of the coordinate system in the defect database pc-space and is given by

A 5 $${\text{n}}_{\mathrm{coh}}^{\left({\mathrm{pc}}_{n}\right)}={\text{V}}_{\mathrm{coh}}^{T}{\text{n}}_{\mathrm{coh}}^{\left(\mathrm{pc}\right)}.$$

The matrix **V**_coh_ is obtained from the singular value decomposition of the covariance matrix **R**^(pc)^_coh_ of vectors **n**^(pc)^_coh_

A 6 $${\text{R}}_{\mathrm{coh}}^{\left(\mathrm{pc}\right)}={\text{V}}_{\mathrm{coh}}{\text{D}}_{\mathrm{coh}}{\text{V}}_{\mathrm{coh}}^{T},$$

where the diagonal matrix **D**_coh_=diag{*d*_coh,1_,…,*d*_coh,*N*_*s*__} contains the eigenvalues of **R**^(pc)^_coh_.

Because all operations are linear, then from the noise model (5.6) it follows that each component of the noise vector **n**^(*pc_n_*)^_coh_ is represented by the linear combination of independent normally distributed random variables. Moreover, it is easy to check that the covariance matrix ${\text{R}}_{\mathrm{coh}}^{\left({\mathrm{pc}}_{n}\right)}$ of vectors **n**^(*pc_n_*)^_coh_ has diagonal form

A 7 $${\text{R}}_{\mathrm{coh}}^{\left({\mathrm{pc}}_{n}\right)}=\frac{1}{N_{\mathrm{coh}}-1}{\text{D}}_{\mathrm{coh}},$$

where *N*_coh_ is the number of noise vectors **n**^(pc)^_coh_. This expression shows that the components of the noise vector ${\text{n}}_{\mathrm{coh}}^{\left({\mathrm{pc}}_{n}\right)}$ now represent independent normally distributed random variables, $n_{\mathrm{coh},i}^{\left({\mathrm{pc}}_{n}\right)}∼N(0,d_{\mathrm{coh},i})$, with the variances equal to the eigenvalues *d*_coh,*i*_, $i=1\ldots N_{s}$.

The noise probability density function *ρ*_*n*_(**n**^(pc)^_coh_) in the defect class pc-space represents the multivariate normal distribution and, using relationship (A 5), can be written as

A 8 $$\rho_{n}({\text{n}}_{\mathrm{coh}}^{\left(\mathrm{pc}\right)})=\frac{1}{\sqrt{\det(2\pi {\text{D}}_{\mathrm{coh}})}}\exp\left[-\frac{1}{2}{\text{n}}_{\mathrm{coh}}^{(\mathrm{pc})T}{\text{V}}_{\mathrm{coh}}{\text{D}}_{\mathrm{coh}}^{-1}{\text{V}}_{\mathrm{coh}}^{T}{\text{n}}_{\mathrm{coh}}^{\left(\mathrm{pc}\right)}\right].$$

If the noise pc-space is normalized with respect to the standard deviations $\sqrt{d_{\mathrm{coh},i}}$

A 9 $${\tilde{\text{n}}}_{\mathrm{coh}}^{\left({\mathrm{pc}}_{n}\right)}=\sqrt{{\text{D}}_{\mathrm{coh}}^{-1}}{\text{n}}_{\mathrm{coh}}^{\left({\mathrm{pc}}_{n}\right)},$$

then each component ${\tilde{n}}_{\mathrm{coh},i}^{\left({\mathrm{pc}}_{n}\right)}$ of the vector ${\tilde{\text{n}}}_{\mathrm{coh}}^{\left({\mathrm{pc}}_{n}\right)}$ in the normalized noise pc-space has the standard normal distribution ${\tilde{n}}_{\mathrm{coh},i}^{\left({\mathrm{pc}}_{n}\right)}∼N(0,1)$. Therefore, the noise probability density function $\rho_{n}({\tilde{\text{n}}}_{\mathrm{coh}}^{\left({\mathrm{pc}}_{n}\right)})$ in the normalized noise pc-space is given by

A 10 $$\rho_{n}({\tilde{\text{n}}}_{\mathrm{coh}}^{\left({\mathrm{pc}}_{n}\right)})=\frac{1}{{\left(2\pi \right)}^{N_{s}/2}}\;\exp\left[-\frac{1}{2}{\left|{\tilde{\text{n}}}_{\mathrm{coh}}^{\left({\mathrm{pc}}_{n}\right)}\right|}^{2}\right].$$

Expression (A 10) provides an efficient way to calculate the defect characterization parameters. According to the discussion in §4, the characterization result corresponds to the point on the *d*-manifold with the highest probability. Formula (A 10) shows that the noise distribution in the normalized noise pc-space is isotropic and the maximum probability point corresponds to the smallest distance from the measurement point to the *d*-manifold in this space. Therefore, the characterization result can be calculated as a projection of the measurement point onto the *d*-manifold in the normalized noise pc-space. In this case, the method described in appendix Aa can be used; however, all vectors have to be transformed into the normalized noise pc-space using expressions (A 5) and (A 9) first. Alternatively, expression (A 8) shows that this projection can also be performed in the defect class pc-space, but in this case a different metric, corresponding to the distance in the normalized noise pc-space, must be used.

Finally, it is noted that, similarly to the defect *d*-manifold, the noise distribution ${\text{n}}_{\mathrm{coh}}^{\left({\mathrm{pc}}_{n}\right)}$ in the noise pc-space is described by the relatively small number of the first largest components, so the noise variances *d*_coh,*i*_→0 as i→∞. Therefore, in order to avoid large errors in calculation of distances in the normalized noise pc-space, vectors ${\text{n}}_{\mathrm{coh}}^{\left({\mathrm{pc}}_{n}\right)}$ have to be truncated using some threshold before applying scaling (A 9).
